# Altered MicroRNA Expression Is Associated with Tumor Grade, Molecular Background and Outcome in Childhood Infratentorial Ependymoma

**DOI:** 10.1371/journal.pone.0158464

**Published:** 2016-07-08

**Authors:** Magdalena Zakrzewska, Wojciech Fendler, Krzysztof Zakrzewski, Beata Sikorska, Wiesława Grajkowska, Bożenna Dembowska-Bagińska, Iwona Filipek, Łukasz Stefańczyk, Paweł P. Liberski

**Affiliations:** 1 Department of Molecular Pathology and Neuropathology, Medical University of Lodz, Lodz, Poland; 2 Department of Biostatistics and Translational Medicine, Medical University of Lodz, Lodz, Poland; 3 Department of Neurosurgery, Polish Mother Memorial Hospital Research Institute, Lodz, Poland; 4 Department of Pathology, Children’s Memorial Health Institute, Warsaw, Poland; 5 Department of Experimental and Clinical Neuropathology, M. Mossakowski Medical Research Centre, Polish Academy of Sciences, Warsaw, Poland; 6 Department of Pediatric Oncology, Children's Memorial Health Institute, Warsaw, Poland; 7 Third Municipal Hospital in Lodz, Lodz, Poland; University Hospital of Navarra, SPAIN

## Abstract

**Background:**

Ependymal tumors are the third most common group of brain tumors in children, accounting for about 10% of all primary brain neoplasms. According to the current WHO classification, they comprise four entities with the most frequent ependymoma and anaplastic ependymoma. The most of pediatric tumors are located within the posterior fossa, with a tendency to infiltrate the vital brain structures. This limits surgical resection and poses a considerable clinical problem. Moreover, there are no appropriate outcome prognostic factors besides the extent of surgical resection. Despite definition of molecular subgroups, the majority of childhood ependymomas present a balanced genome, which makes it difficult to establish molecular prognostic factors.

**Methods:**

The purpose of our study was to explore whether miRNA expression could be used as prognostic markers in pediatric infratentorial ependymomas. We also performed a mRNA expression pattern analysis of *NELL2* and *LAMA2* genes, with immunohistochemical illustrations of representative cases. The miRNA and mRNA expression was measured in 53 pediatric infratentorial ependymomas using a real-time quantitative PCR.

**Results:**

Three miRNAs were shown to efficiently differentiate between grade II and III ependymomas: miR-17-5p, miR-19a-3p, and miR-106b-5p. Survival analysis showed that the probabilities of overall (p = 0.036) and event-free survival (p = 0.002) were reduced with higher than median miRNA expression levels of miR-17-5p. Using multivariate analysis adjusted for patient's age, sex, tumor grade and localization, we showed statistically significant associations with event-free survival (p = 0004) and borderline statistical significance with overall survival (p = 0.057) for miR-17-5p. Correlation analysis of miR-19a, miR-17-5p, miR-106b revealed that their expression levels were significantly correlated with *EZH2* expression, suggested marker of PFA ependymomas. Furthermore, lower expression level of *LAMA2* mRNA was shown to be associated with an increased risk of death in covariate-adjusted analyses.

**Conclusions:**

Our data provide a better understanding of pediatric ependymoma and suggests the presence of plausible molecular biomarkers connected with the outcome.

## Introduction

Ependymomas accounts for about 10% of all primary central nervous system tumors in the pediatric population. According to the current World Health Organization (WHO) 2007 classification of brain tumors, ependymal tumors comprise four entities with the most frequent ependymomas, WHO grade II, and anaplastic ependymomas, WHO grade III [1].

Recently, on the basis of large scale analysis nine molecular subgroups, three in each anatomical compartment were also proposed [2,3,4].

Nevertheless, in children below 5 years of age nearly 70% of ependymomas develop within the posterior fossa and show balanced genome features [4]. It is strongly suggested that the most relevant analyses in tumors with such a “silent” molecular background are connected with epigenetic and regulatory studies. This is motivated by the fact that the activation of a number of pivotal genes could be altered by RNA (microRNA) interference and/or methylation-associated silencing. Nowadays, the importance of epigenetic processes in pediatric brain cancer tumorigenesis continues to increase. This is reflected in analyses showing epigenetic reprogramming connected with *H3F3A* mutations as essential for pediatric glioblastoma formation, as well as the observation that *DNMT3B* upregulation, correlated with the largest cluster of miRNA genes (*C19MC*) amplification due to *TTYH1* fusion, is critical in embryonal tumors with multilayered rosettes (ETMR) [5,6]. A recent study describing CIMP-positive subtype of ependymoma highlighting the role of such processes also in those tumors [7].

Until now, the analysis of miRNA and its key role in regulating gene expression has not been studied in depth in pediatric ependymomas, despite the increasing interest in their role in the formation of other brain tumors (glioma, medulloblastoma) [8–10].

MiRNAs are a type of small non-coding RNAs acting as regulators of gene expression, which connects them with multiple oncogenic processes, such as the initiation of tumor growth and the progression and formation of metastases [11]. Considering this, it is hardly surprising that recent reports have shown the usefulness of miRNA evaluation for molecular diseases stratification and finding a new tools for targeted cancer therapy [12–16].

In the current study we analyzed miRNA expression in a representative group of Polish pediatric infratentorial ependymomas and considered their connection with histological, molecular and clinical data.

## Materials and Methods

### Patient samples

A total of 53 children with infratentorial ependymomas were included into this study. All patients were under 18 years of age (ranging from 6 months to 15 years at the time of surgery), with a median age of 5 years. The protocol of the study was approved by the Bioethical Committee at the Medical University of Lodz (permit No: RNN/175/15/KE). Written informed consent has been obtained from all examined individuals. Written informed parental consent was obtained for all patients under 16. For children below 16 years of age the consent was given by parents, for older (16–17) informed consent was obtained from both parents and child, according to the Polish law.

The group was comprised of 36 cases of tumors classified as WHO grade II and 17 cases classified as WHO grade III ependymoma. Histopathological grading was conducted on the basis of WHO classification and proposed recently novel diagnostic criteria [1,17]. Molecular subtypes of infratentorial tumors were defined on the basis of *EZH2* gene expression [18].

For five patients the biological material from primary and recurring tumors was available. The tumors have the same histopathological features and classification. All data were processed and stored in compliance with the Helsinki Declaration. The clinical data of the samples used in this study are presented in Table 1.

**Table 1:** Clinicopathologic features of infratentorial ependymoma.

Variable	Number	%
Age [years]		
<4	26	49%
4–16	26	49%
>16	1	2%
Gender		
Male	35	66%
Female	18	34%
WHO classification		
grade II	36	68%
grade III	17	32%
Recurrence	18	34%
**Total**	**53**	**100%**

### RNA extraction and cDNA synthesis

Total RNA, including miRNA, was extracted from tissue fractions stabilized in RNAlater and stored at -80°C until further processing. Samples were homogenized in QIAzol Lysis Reagent, using a miRNeasy Mini Kit (Qiagen GmbH, Hilden, Germany) in accordance with the manufacturer’s protocol. Briefly, for a period of 5 min, 700 μl QIAzol Lysis Reagent was added to the sample, and incubated at room temperature. After adding 140 μl of chloroform, the samples were shaken vigorously for 15 s, incubated at room temperature, and centrifuged for 15 min at 4°C. The upper aqueous phase was mixed with 1.5 times its volume of 100% ethanol, transferred to a spin column, centrifuged, washed, and eluted in 30 μl RNase-free water. Samples were used for cDNA synthesis, using the miScript ІІ RT Kit in accordance with the manufacturer’s recommendations (Qiagen GmbH, Hilden, Germany). 500 ng of each total RNA sample was used for cDNA synthesis with 5xHiFlex Buffer in 20 μl reverse-transcription reaction master mixes. For selective conversion of 250 ng of each mature miRNA into cDNA the reverse-transcription reaction mix with 5xHiSpec Buffer was prepared (20 μl). The reaction mixtures were incubated for 60 min at 37°C, followed by denaturation for 5 min at 95°C. Each cDNA was further diluted according to the protocol and stored at -20°C until use.

### MicroRNA profiling

Initial screening was done for 11 posterior fossa tumors and was focused on identifying of miRNAs with plausible value of risk stratification for patients with ependymomas. Analyses were conducted with using the miScript miRNA PCR Array, Human Brain Cancer miRNA PCR Array (MIHS-108Z, SABiosciences, Qiagen). The Rotor-Disc format wells were distributed as follows: 84 miScript Primer Assays, replicate *C*. *elegans* miR-39 assay for internal array data normalization, snoRNA/snRNAs miScript PCR controls (SNORD61, SNORD68, SNORD72, SNORD95, SNORD96A, RNU6B/RNU6-2), replicate reverse transcription reaction controls (miRTC), and positive PCR control (PPC) (http://www.sabiosciences.com/mirna_pcr_product/HTML/MIHS-108Z.html). This allowed for assessing the expression of 84 miRNA sequences most relevant to brain tumors. All reactions were performed using SYBR Green-based real-time PCR with the miScript SYBR Green PCR Kit (Qiagen GmbH, Hilden, Germany) on the Rotor Gene 6000 instrument (Qiagene-Corbett Life Science, Sydney, Australia). The miScript cycling conditions were as follows: an initial activation at 95°C for 15 min, followed by 40 cycles of 94°C for 15 s, at 55°C for 30 s, and at 70°C for 30 s.

### MicroRNA validation

The validation was used to confirm the differences and evaluate whether such miRNAs expression could also be associated with histological, molecular and clinical data. Differentially expressed miRNAs were selected for expression measurements in an independent validation group which consisted of 42 cases of infratentorial ependymomas: hsa-miR-200a-3p (miScript Primer Assay no. MS00003738), hsa-miR-19a (miScript Primer Assay no. MS00003192), hsa-miR-106b (miScript Primer Assay no. MS00003402), hsa-miR-17-5p (miScript Primer Assay no. MS00029274). One miRNA of stable expression, has-miR-9 was used as an internal control (miScript Primer Assay no. MS00006510). Correlation analysis of RT-qPCR and array expression values was carried out using SYBR Green-based real-time PCR with the miScript SYBR Green PCR Kit (Qiagen GmbH, Hilden, Germany) on the Rotor Gene 6000 instrument (Qiagene-Corbett Life Science, Sydney, Australia). The PCR reactions for each assay were run in duplicate and the results were averaged over those analyzes. The normalized relative expression level of the genes of interest was calculated according to the dCt method.

### Gene expression analysis

During experiment the relative changes in gene expression was analyzed for *LAMA2* (TaqMan^®^ Gene Expression Assay Hs01124081_m1), *NELL2* (TaqMan^®^ Gene Expression Assay Hs00196254_m1) and *EZH2* (TaqMan^®^ Gene Expression Assay Hs01016789_ml) genes with *GAPDH* (TaqMan^®^ Gene Expression Assay Hs02758991_g1) as the reference. All reactions were prepared with TaqMan^®^ Universal PCR Master Mix (cat. no. 4369016, Applied Biosystems, UK) and performed on a Rotor Gene 6000 instrument (Qiagene-Corbett Life Science, Sydney, Australia). PCR reactions for each assay were run in duplicate and the results were an averaged over the two analyses.

### Immunohistochemistry

To determine the immunohistochemistry of representative cases, 5 μm thick sections of formalin-fixed and paraffin-embedded tissue were used with the previously recommended antibodies: anti-NELL2 (ab80885, Abcam, dilution 1:250), and anti-LAMA2 (H00003908-M01, Abnova, dilution 1:1000) [19]. The epitope retrieval method included citrate buffer (pH 9) and overnight heating at 100°C in a water bath. For visualization we used the Dako EnVision + System-HRP (DAB) for Use with Mouse Primary Antibodies (K4007) for LAMA2, and Dako EnVision+System-HRP (DAB) for Use with Rabbit Primary Antibodies (K4011) for NELL2 detection.

### Statistical analysis

Medians with 25–75 percentile values were used to describe patient’s age, due to its non-normal distribution in the studied group. Average and standard deviations were used to represent expression values in the compared groups. Student’s t-test was used to compare expression data in both the profiling experiment and the validation cohort. An adjustment for multiple hypothesis testing was carried out using the Benjamini-Hochberg procedure in the profiling experiment. Paired analyses between primary and relapse samples were performed using the Student’s t-test for paired samples. Principal component analysis was used to visualize paired miRNA expression profiles. Survival analysis was performed using the log-rank test and multivariate Cox’ proportional hazards regression. Hazard ratios (HR) were calculated per 1 dCt, which represented a twofold decrease of miRNA expression. Statistical significance was assumed for p-values ≤ 0.05.

## Results

### A subset of miRNAs is differentially expressed in infratentorial ependymomas of childhood

**Fig 1:**
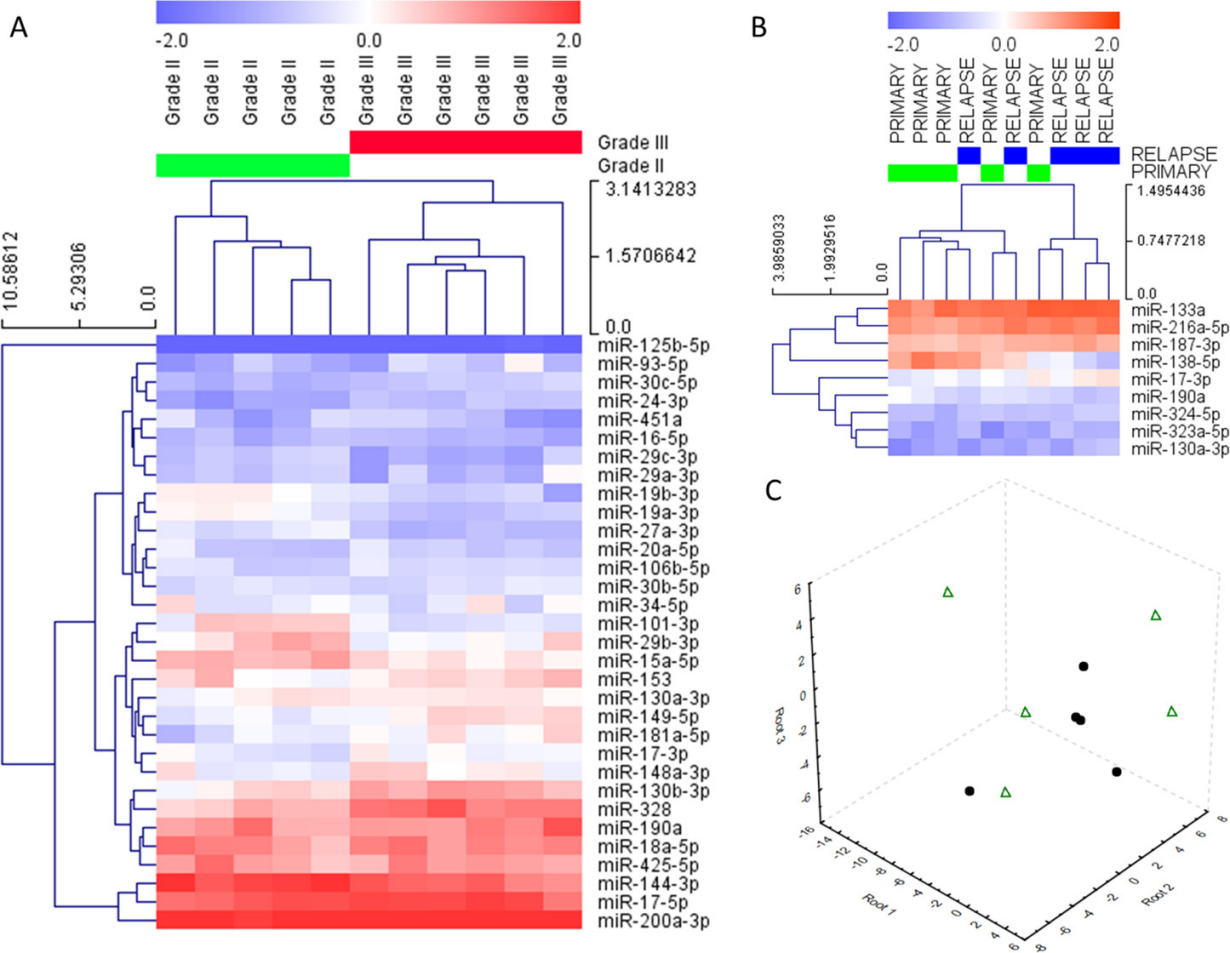
Profiling of miRNA in infratentorial ependymomas. (A) Hierarchical clustering heatmap of miRNAs differentially expressed in grade II and grade III ependymomas. (B) Subset of primary and recurrent lesions obtained from the same patient showed no significant changes in miRNA expression. (C) miRNA expression showed weak association with the stage of disease; a circle–primary tumors, a triangle–recurrence tumors.

Out of 84 profiled miRNAs, 32 showed differential expression between grade II and grade III posterior fossa tumors (S1 Table, Fig 1A). Grade II ependymomas showed lower expression of differentially expressed miRNAs than WHO grade III ependymomas (S2 Table).

Spearman's rank-correlation coefficient was used to analyze the correlations between differentially expressed miRNAs and *EZH2* gene status. As it was recently suggested, posterior fossa ependymomas expressing *EZH2* may have the same epigenetic signature as the PFA tumors connected with CpG island methylator phenotype. Since we were unable to perform methylation arrays the next more appropriate solution was the evaluation of *EZH2* expression in analyzed here cases [18]. Gene expression was considered as dichotomous variable, with individual expression levels coded as over- or underexpressed with respect to median expression. The values of correlation between differentially expressed miRNAs and *EZH2* expression in posterior fossa ependymomas are listed in Table 2.

**Table 2:** Correlation between miRNA and *EZH2* expression in profiled cases.

miRNA	r	p-value
miR-107	0.8716	0.002
miR-216a-5p	0.8472	0.004
miR-130b-3p	0.8284	0.006
miR-17-5p	0.8205	0.007
miR-137	0.7937	0.011
miR-187-3p	0.7853	0.012
miR-218-5p	0.7647	0.016
miR-217	0.7559	0.018
miR-106b-5p	0.7528	0.019
miR-20a-5p	0.7246	0.027
miR-130a-3p	0.7193	0.029
miR-182-5p	0.7175	0.030
miR-323a-5p	0.6972	0.037
miR-19b-3p	0.6787	0.044
miR-221-3p	0.6638	0.051
miR-19a-3p	0.6443	0.061
miR-96-5p	0.6186	0.076
miR-183-5p	0.6074	0.083
miR-200a-3p	0.5999	0.087
miR-138-5p	0.5992	0.088
miR-9-3p	-0.5999	0.088
miR-335-5p	0.5857	0.098
miR-31-5p	-0.5405	0.133
miR-222-3p	0.5313	0.141
miR-9-5p	-0.5309	0.141
miR-148a-3p	0.5304	0.142

r–correlation coefficient

Within this profiling group there were five cases of paired primary and recurrent tumors. Paired analysis of primary and recurrent lesions obtained from the same patients showed no clear tendency of changes in miRNA expression connected with recurrence of disease (Table 3, Fig 1B). The expression profile seemed to be connected with intraindividual variability rather than with the tumor’s stage of disease (Fig 1C).

**Table 3:** dCt values of miRNAs in primary and recurrent lesions.

miRNA	Primary		Relapse		p-value	FDR
	Average	SD	Average	SD		
miR-190a	0.28	0.22	0.18	0.19	0.0165	0.3903
miR-216a-5p	1.35	0.09	1.55	0.06	0.0176	0.3903
miR-187-3p	1.05	0.08	1.23	0.09	0.0196	0.3903
miR-130a-3p	-0.39	0.08	-0.15	0.17	0.0260	0.3903
miR-324-5p	-0.12	0.12	0.12	0.10	0.0287	0.3903
miR-17-3p	0.48	0.10	0.64	0.15	0.0316	0.3903
miR-133a	1.62	0.14	1.73	0.11	0.0397	0.3903
miR-323a-5p	-0.44	0.27	-0.04	0.13	0.0402	0.3903
miR-138-5p	1.12	0.60	0.60	0.62	0.0418	0.3903

The dCt is given in the logarithmic scale therefore higher dCt values indicate a lower normalized target gene expression. FDR–false discovery rate; SD–standard deviation

### miRNAs expression on validation cohort is associated with tumor grade, molecular subtype and prognosis

**Fig 2:**
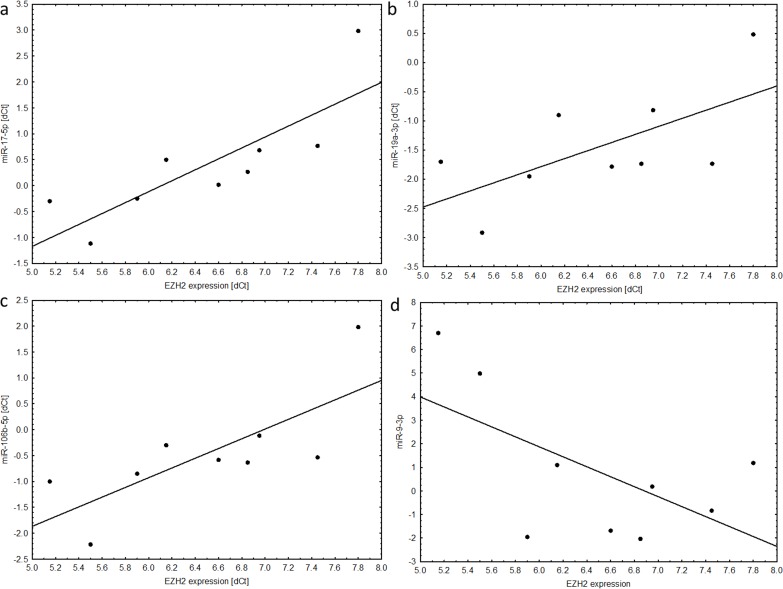
miRNA relative expression in the validation cohort of grade II and grade III ependymoma. (A) miR-17-5p (B) miR-200a (C) miR-106b (D) miR-19a.

During validation of profiling data we obtained convergent results of miRNA expression. Similarly to the original dataset, lower expression of miRNAs was noted for grade II tumors (Fig 2).

Expression of miR-17-5p, miR-19a and miR-106b was significantly lower in grade II than in grade III infratentorial tumors. No significant differences were noted for miR-200a-3p. Lower dCt scores were observed in grade III tumors what represented the higher expression of selected miRNAs (Table 4).

**Table 4:** dCt values of grade predictors in validation cohort.

miRNA	Family	Cluster	Grade II		Grade III		p-value
			Avg	SD	Avg	SD	
miR-200a-3p	mir-200	miR-200bc/429	9.20	2.49	8.00	1.50	0.075
miR-17-5p	mir-17	miR-17/92	1.74	1.87	0.53	1.47	0.038
miR-19a	mir-19	miR-17/92	2.65	2.70	-0.05	0.78	<0.001
miR-106b	mir-17	miR-106/25	2.15	1.91	0.18	0.51	<0.001
**GENE**							
*NELL2*			6.27	1.75	5.91	2.58	0.659
*LAMA2*			9.20	2.49	8.00	1.50	0.075

SD–standard deviation

**Fig 3:**
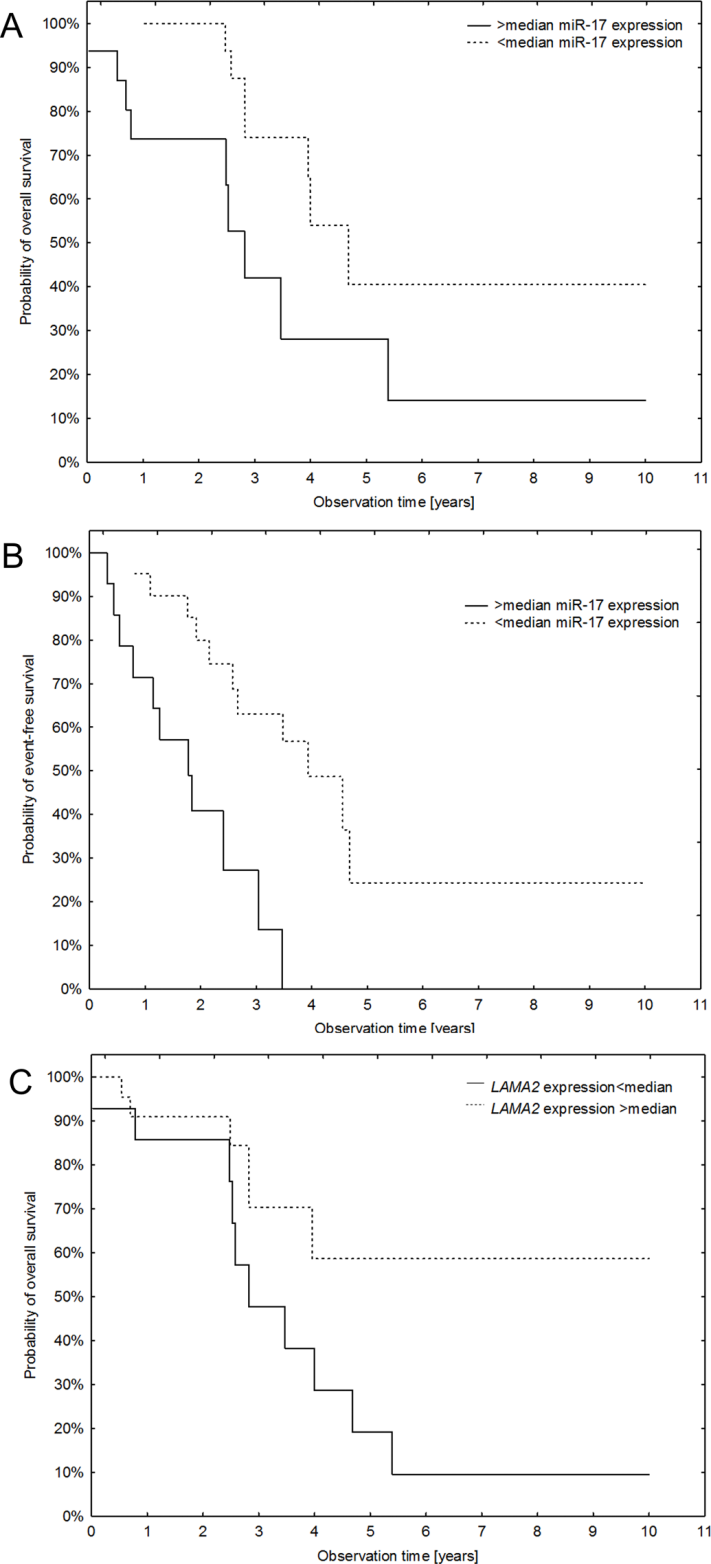
Survival analysis of patients with ependymoma. (A) Kaplan-Meier curves of overall survival depending on miR-17-5p expression dichotomized using median expression levels, p = 0.036. The probability of death was significantly higher in samples with higher expression of miR-17-5p. (B) Differences in event-free survival between samples with high and low expression of miR-17-5p, p = 0.002. The probability of death was significantly higher in samples with higher expression of miR-17-5p. (C) Differences in overall survival between samples with high and low expression of *LAMA2*, p = 0.069. The probability of death was higher in samples with lower expression of *LAMA2*.

Survival analysis showed that the probabilities of overall (HR = 2.93, 95%CI = 1.07–8.01, p = 0.036) and event-free survival (HR = 4.77, 95%CI = 1.79–12.76, p = 0.002) were reduced with higher than median miRNA expression levels of miR-17 (Fig 3A and 3B).

None of the other three miRNAs (miR-200a, miR-19a, miR-106b) showed significant associations with overall or event-free survival (all p-values >0.15).

Also higher *LAMA2* expression was associated with poorer prognosis, although the effect did not reach statistical significance (HR = 0.39, 95%C = 0.14–1.07, p = 0.069) (Fig 3C). No significant associations were detected between expression of *LAMA2* and event-free survival or *NELL2* with either overall or event-free survival probabilities (all p-values >0.15).

After adjustment for patients’ age, sex, tumor grade and localization, higher expression of miR-17-5p was shown to be associated with increased risk of death at borderline statistical significance (HR = 3.26, 95%CI = 0.96–11.04, p = 0.057) and significantly increased risk of relapse (HR = 4.96, 95%CI = 1.67–14.68, p = 0004). Multivariate analysis of other miRNAs did not revealed any significant associations with overall or event-free survival (Table 5).

**Table 5:** Multifactorial analysis of molecular prognostic factors.

	**Overall Survival**			
**miRNA/Gene**	HR (95%CI)*	**p-value**	**HR (95%CI)**^**$**^	**p-value**
dCt miR-17-5p	2.93 (1.07–8.01)	0.036	3.26 (0.96–11.04)	0.057
dCt miR-200a-3p	1.44 (0.53–3.93)	0.474	1.41 (0.50–3.98)	0.516
dCt miR-19a	1.23 (0.46–3.31)	0.678	1.00 (0.29–3.39)	0.999
dCt miR-106b	1.46 (0.54–3.97)	0.455	0.94 (0.23–3.76)	0.927
*NELL2*	1.33 (0.50–3.55)	0.574	1.40 (0.49–4.00)	0.530
*LAMA2*	0.39 (0.14–1.07)	0.069	0.41 (0.13–1.35)	0.144
	**Event-Free Survival**			
**miRNA/Gene**	HR (95%CI)*	**p-value**	**HR (95%CI)**^**$**^	**p-value**
dCt miR-17-5p	4.78 (1.79–12.76)	0.002	4.96 (1.67–14.68)	0.004
dCt miR-200a-3p	1.75 (0.75–4.10)	0.194	1.73 (0.73–4.07)	0.211
dCt miR-19a	1.47 (0.63–3.42)	0.373	1.47 (0.51–4.25)	0.784
dCt miR-106b	1.31 (0.56–3.06)	0.527	0.89 (0.26–3.01)	0.855
*NELL2*	1.95 (0.83–4.62)	0.127	2.07 (0.84–5.12)	0.113
*LAMA2*	0.55 (0.23–1.28)	0.166	0.59 (0.34–1.45)	0.252

Hazard ratios (HR) denote risk of death associated with lower expression level observed in the studied group. CI, confidence interval

*, unadjusted values

^$^, values adjusted for sex, age, grade and tumor location.

p≤ 0.05.

**Fig 4:**
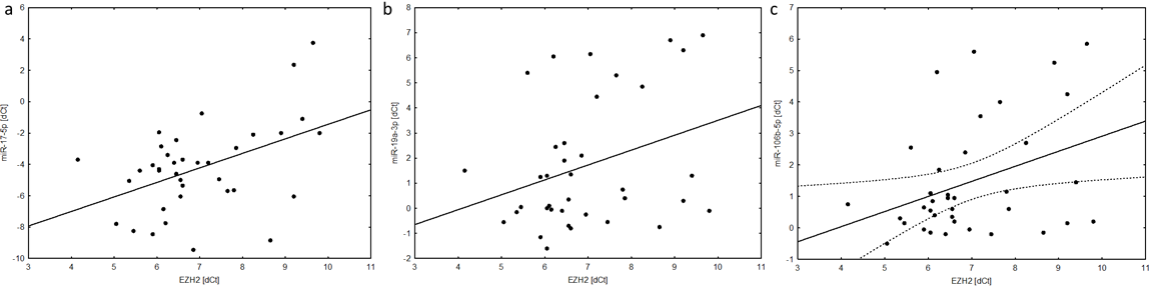
Correlation between miRNA and *EZH2* dCt values in validation group. (A) miR-17-5p vs. *EZH2*. (B) miR-19a vs. *EZH2*. (C) miR-106b vs. *EZH2*.

Correlation analysis on the basis of Spearman rank correlation test revealed statistically important connection of outcome-associated miR-17-5p with *EZH2* expression (r = 0.45, p = 0.005) what indicated for PFA tumors as miR-17-5p related cases. Moreover positive correlation for miR-106b and a considerable trend toward significance in case of miR-19a was noted in relation to *EZH2* expression (r = 0.36, p = 0.027 and r = 0.32, p = 0.054 respectively) what altogether indicated for miR-17/92 and miR-106/25 cluster families as significant for PFA ependymal tumors (Fig 4). Correlation for miR-200a-3p did not reach statistical significance (p = 0.458).

### Correlation of *LAMA2* and *NELL2* expression with patient’s outcome

In our analysis average dCt values for *LAMA2* and *NELL2* genes expression were higher in grade III ependymomas what indicated that more aggressive tumors showed lower expression values. The probability of death was significantly higher in samples with lower expression of *LAMA2* although observed differences were not statistically significant (p = 0.069) but revealed a certain trend toward significance (Fig 3C, Table 4).

No significant associations were detected between expression of *LAMA2* and event-free survival or *NEL*L2 with either overall or event-free survival probabilities (all p-values >0.15).

**Fig 5:**
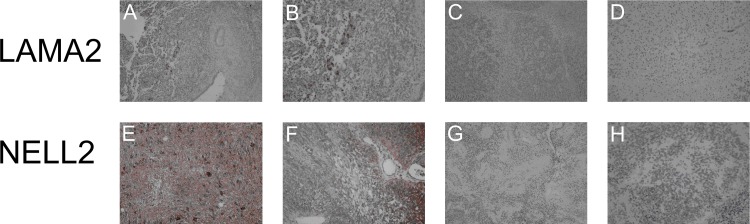
LAMA2 and NELL2 immunoreactivity on ependymoma representative slides. (A) LAMA2 positive staining showing focal cytoplasmic immunoreactivity of tumour cells, magnification 100X. (B) LAMA2 positive staining showing focal cytoplasmic immunoreactivity of tumour cells, magnification 200X. (C) LAMA2 negative staining, magnification 100X. (D) LAMA2 negative staining showing immunoreactivity in the endothelial cells of vessels, magnification 200X. (E) NELL2 positive staining with strong widespread cytoplasmic immunoreactivity of tumour cells, magnification 100X. (F) NELL2 positive staining with strong widespread cytoplasmic immunoreactivity of tumour cells, magnification 100X. (G) NELL2 positive staining with cytoplasmic immunoreactivity of tumour cells, magnification 200X.

Immunohistochemical examination performed on representative cases showed immunoreactivity in tumor slides with altered mRNA expression (Fig 5).

Our analysis did not revealed correlation between selected miRNAs and *LAMA2* and *NELL2* genes expression. Prediction of target interactions with using current databases (http://mirtarbase.mbc.nctu.edu.tw/index.php) also did not confirmed potential miRNA-target interactions (S3 Table) [20].

## Discussion

The prognostic value of WHO classification in particular cases of ependymomas still remains controversial, and current criteria do not link the grade and course of the disease in a consistent way [21,22]. Additionally, in the current literature there are analyses that pointed out strong influence of individual histopathological evaluation for results between grade and outcome [17]. For such reasons a grading system of ependymomas is still under discussion by clinicians and scientists alike. Regardless of that the big effort has been made to define molecular background of ependymoma of different anatomical location and age group [2,3,4]. However classic tumors classification provides still worthwhile information and clinical usefulness of achievements in molecular subgrouping of ependymoma needs further confirmation. Moreover the question of new biomarkers considered for diagnosis and risk stratification in children with ependymomas is still topical. Recently, as an accessible and non-invasive molecular tool has been undertaken miRNAs, but its implications for ependymomas biology are still poorly understood [8–10].

According to that we perform miRNA profiling study on the group of childhood infratentorial ependymomas of different grade, molecular background and outcome. We revealed presence of 32 differentially expressed miRNAs when comparing grade II and grade III tumors. The selected on the basis of bioinformatic analysis miR-17-5p, miR-19a, and miR-106b showed a statistical significance in relation to tumor grade and molecular subtype also on validation cohort, with higher expression levels in grade III ependymomas.

Such an observation is in concordance with previous result highlighting global up- rather than down-regulation of miRNAs when comparing tumor and normal samples [9]. The results of the first investigation, comprising eight samples of ependymomas, were published in Birks et al. survey. After comparison of tumor and normal tissue, the authors indicated both up-regulated molecules (miR-34c, miR-34b, miR-200b, miR-200a, miR-483, miRNA-142-5p) as well as down-regulated ones (miR-137, miR-138, miR-124a, miR-181d, miR-193b). They pointed out the need for further analyses of miR-129, miR-142-5p, and miR-25 as the most specific for pediatric brain tumors as a whole [8].

Novelty of our analysis is strong suggestion of the miR-17/92 cluster family as important for ependymoma biology. Selected here miR-19a, miR-17-5p and miR-106b belong to the miR-17/92 cluster family known as Oncomir-1 or to their paralog, miR-106/25 [23–25]. The overexpression of those clusters members have documented role in tumorigenesis and has been reported in cancer development [12,16,26–30].

MiR-17-5p, connected with event free and overall survival in analyzed here cases have important role in tumorigenesis, mainly due to their impact on cell proliferation, stem cell maintenance, as well as developmental processes [25,26,30]. On the basis of in vitro studies, a both pro- and antiproliferative effect has been described for this molecule, and this plurality allows miR-17-5p to act as either a tumor suppressor or oncogene, depending on the cellular context [30]. This ambiguous functionality has resulted in finding of opposite clinical significance observed in different types of cancer [12,14,16,26,31]. For example in brain tumors of neuroepithelial origin protective function of miR-17-5p was described [16,28,30]. Such observations may be a consequence of miR-17-5p acting as a component of a large genetic network modulated by transcriptional factors, cyclin-dependent kinases and having caused tissue-, organ- and even tumor-specific expressions due to its unexpected cellular targets [30]. The role of mir-17-5p in brain tumors is still not well documented but our observation indicated that poor prognosis of overexpressed patient groups could be a phenomenon restricted not only for hepatocellular or ovarian cancer [9,16,23,24,27].

The noted here higher miRNAs expression in anaplastic ependymomas allowed to predict a shorter time period to relapse and was connected with decreased survival in our cohort. Such an observation could be perceived as a valuable finding until the troublesome histological grading of ependymomas occurred. In the analyses published thus far, the relationship between outcome and grading remains still confusing [17,29,32,33]. Therefore the differentiation between grade II and grade III ependymomas seems to be related not only to histological but also to molecular factors and in ependymomas it has special importance until tumor grading has ambiguous utility. Our analyses showed also correlation of outcome-related miRNAs with *EZH2* gene expression levels, both in profiling and validation group. *EZH2* was recently suggested as the marker of a poor prognosis in children with infratentorial ependymoma. Because of its connection with epigenetic regulation through PRC complex up-regulation of *EZH2* could be considered as the discriminator of PFA ependymomas [18]. Such correlation could indicate for miRNA as plausible additional molecular factor of poor progosis in children with infratentorial ependymomas.

Together with miRNA analysis, we conducted expression studies of *LAMA2* and *NELL2* genes, which have been suggested as a possible prognostic factors for children with posterior fossa ependymomas [19,21]. In our study an increased expression of *LAMA2* indicated for a better overall survival rate of the patients without significant connection with tumor grade.

*LAMA2* is expressed in most human tissues and several other regions of the brain, including the meninges and choroid plexus. This is an extracellular protein involved in brain development mainly by affecting cellular migration and differentiation [34]. This component of the extracellular matrix defect is widespread in human cancer, and tumors with lower expression of laminins represent more aggressive subtypes with a significantly worse survival outcome [35]. Unfortunately, there are limited studies concerning the role of *LAMA2* in brain tumors, although it has been postulated that this is an element of the differentiation niche of glioma initiating cells and considered responsible for therapeutic resistance and the recurrence of a subset of glial tumors [36]. Such an observation points to an important role of the extracellular matrix and cell adhesion in brain tumors progression. Single transcriptome analyses indicated the linked to a worse prognosis upregulation of *LAMA2* in PFA ependymomas [19, 21]. Here we have shown an opposite effect of *LAMA2* expression status on patient’s outcome. On the other side, we confirmed correlation of *EZH2* up-regulated cases with selected here outcome-related miRNAs. In addition, similar as Lourdusamy et al. in their ependymoma study showing a link between the miR-29a/c family and *LAMA2* expression, we indicated for plausible functional connection between miR-106b and *EZH2* gene. Such variability need for further elucidation of this phenomenon and establish the methodology and diagnostic usefulness of such candidates for markers in ependymomas. Therefore multifactorial analysis seems to be more accurate in such cases especially when heterogeneity in immunohistochemistry-stained distribution of LAMA2 without correlation with other biomarkers has been observed [10,37].

Our study have shown the clear tendency to increased miRNA expression in higher grades of ependymomas. We have demonstrated a better outcome for patients with miR-17-5p lower expression. Moreover miRNAs expression have been showed to be correlated with *EZH2* expression. On the basis of the literature and our findings, miRNAs seems to be a promising additional candidate for prognostic markers in ependymoma and could be the beginning of targeted miRNA-based treatment in the future. Nevertheless, appropriate disease stratification on the basis of histopathological and molecular biomarkers needs further standardization and optimization.

## Supporting Information

S1 Table Raw data of miRNA expression in the profiling experiment. (XLSX)

S2 Table Expression levels of miRNA in the profiling experiment studied according to the tumor’s subgroups. (XLSX)

S3 Table MiRNAs predicted by miRTarBase to target the *LAMA2* and *NELL2* genes. (DOCX)
