# Supplementary material for: Altered MicroRNA Expression Is Associated with Tumor Grade, Molecular Background and Outcome in Childhood Infratentorial Ependymoma
Source: PLoS One. 2016 Jul 8;11(7):e0158464. doi: 10.1371/journal.pone.0158464 (PMC4938415; doi:10.1371/journal.pone.0158464)
Supplement: S3 Table — (DOCX) [file pone.0158464.s003.docx]

| [**http://mirtarbase.mbc.nctu.edu.tw/index.php**](http://mirtarbase.mbc.nctu.edu.tw/index.php) | [**miRNA**](http://mirtarbase.mbc.nctu.edu.tw/php/search.php?org=hsa&kw=lama2&opt=target&sort=mirna_name&order=desc) | [**Target**](http://mirtarbase.mbc.nctu.edu.tw/php/search.php?org=hsa&kw=lama2&opt=target&sort=gene_symbol&order=desc) |
| --- | --- | --- |
| [MIRT522118](http://mirtarbase.mbc.nctu.edu.tw/php/detail.php?mirtid=MIRT522118) | hsa-miR-297 | NELL2 |
| [MIRT522119](http://mirtarbase.mbc.nctu.edu.tw/php/detail.php?mirtid=MIRT522119) | hsa-miR-3924 | NELL2 |
| [MIRT522120](http://mirtarbase.mbc.nctu.edu.tw/php/detail.php?mirtid=MIRT522120) | hsa-miR-190a-3p | NELL2 |
| [MIRT522121](http://mirtarbase.mbc.nctu.edu.tw/php/detail.php?mirtid=MIRT522121) | hsa-miR-6867-5p | NELL2 |
| [MIRT522122](http://mirtarbase.mbc.nctu.edu.tw/php/detail.php?mirtid=MIRT522122) | hsa-miR-223-5p | NELL2 |
| [MIRT522123](http://mirtarbase.mbc.nctu.edu.tw/php/detail.php?mirtid=MIRT522123) | hsa-miR-567 | NELL2 |
| [MIRT522124](http://mirtarbase.mbc.nctu.edu.tw/php/detail.php?mirtid=MIRT522124) | hsa-miR-769-5p | NELL2 |
| [MIRT522125](http://mirtarbase.mbc.nctu.edu.tw/php/detail.php?mirtid=MIRT522125) | hsa-miR-3185 | NELL2 |
| [MIRT522126](http://mirtarbase.mbc.nctu.edu.tw/php/detail.php?mirtid=MIRT522126) | hsa-miR-4307 | NELL2 |
| [MIRT522127](http://mirtarbase.mbc.nctu.edu.tw/php/detail.php?mirtid=MIRT522127) | hsa-miR-511-3p | NELL2 |
| [MIRT522128](http://mirtarbase.mbc.nctu.edu.tw/php/detail.php?mirtid=MIRT522128) | hsa-miR-5011-5p | NELL2 |
| [MIRT522129](http://mirtarbase.mbc.nctu.edu.tw/php/detail.php?mirtid=MIRT522129) | hsa-miR-6507-5p | NELL2 |
| [MIRT717761](http://mirtarbase.mbc.nctu.edu.tw/php/detail.php?mirtid=MIRT717761) | hsa-miR-5571-3p | NELL2 |
| [MIRT717762](http://mirtarbase.mbc.nctu.edu.tw/php/detail.php?mirtid=MIRT717762) | hsa-miR-4266 | NELL2 |
| [MIRT717763](http://mirtarbase.mbc.nctu.edu.tw/php/detail.php?mirtid=MIRT717763) | hsa-miR-331-3p | NELL2 |
| [MIRT717764](http://mirtarbase.mbc.nctu.edu.tw/php/detail.php?mirtid=MIRT717764) | hsa-miR-6810-3p | NELL2 |
| [MIRT717765](http://mirtarbase.mbc.nctu.edu.tw/php/detail.php?mirtid=MIRT717765) | hsa-miR-6801-3p | NELL2 |
| [MIRT717766](http://mirtarbase.mbc.nctu.edu.tw/php/detail.php?mirtid=MIRT717766) | hsa-miR-6729-3p | NELL2 |
| [MIRT717767](http://mirtarbase.mbc.nctu.edu.tw/php/detail.php?mirtid=MIRT717767) | hsa-miR-4779 | NELL2 |
| [MIRT717768](http://mirtarbase.mbc.nctu.edu.tw/php/detail.php?mirtid=MIRT717768) | hsa-miR-4695-5p | NELL2 |
| [MIRT444720](http://mirtarbase.mbc.nctu.edu.tw/php/detail.php?mirtid=MIRT444720) | hsa-miR-580-3p | LAMA2 |
| [MIRT444721](http://mirtarbase.mbc.nctu.edu.tw/php/detail.php?mirtid=MIRT444721) | hsa-miR-4452 | LAMA2 |
| [MIRT444722](http://mirtarbase.mbc.nctu.edu.tw/php/detail.php?mirtid=MIRT444722) | hsa-miR-183-3p | LAMA2 |
| [MIRT444723](http://mirtarbase.mbc.nctu.edu.tw/php/detail.php?mirtid=MIRT444723) | hsa-miR-539-5p | LAMA2 |
| [MIRT444724](http://mirtarbase.mbc.nctu.edu.tw/php/detail.php?mirtid=MIRT444724) | hsa-miR-4511 | LAMA2 |
| [MIRT444725](http://mirtarbase.mbc.nctu.edu.tw/php/detail.php?mirtid=MIRT444725) | hsa-miR-3133 | LAMA2 |
